# A Monitoring System for Laying Hens That Uses a Detection Sensor Based on Infrared Technology and Image Pattern Recognition

**DOI:** 10.3390/s17061195

**Published:** 2017-05-24

**Authors:** Mauro Zaninelli, Veronica Redaelli, Fabio Luzi, Valentino Bontempo, Vittorio Dell’Orto, Giovanni Savoini

**Affiliations:** 1 Department of Human Sciences and Quality of Life Promotion, Università Telematica San Raffaele Roma, Via di Val Cannuta 247, Rome 00166, Italy; 2 Freelance Certified Infrared Thermal Technician, Oggiono (LC) 23848, Italy; vereda@tin.it; 3 Department of Veterinary Medicine, Università degli Studi di Milano, Via Celoria 10, Milan 20133, Italy; fabio.luzi@unimi.it; 4 Department of Health, Animal Science and Food Safety (VESPA), Università degli Studi di Milano, Via Celoria 10, Milan 20133, Italy; valentino.bontempo@unimi.it (V.B.); vittorio.dellorto@unimi.it (V.D.); giovanni.savoini@unimi.it (G.S.)

**Keywords:** infrared sensors, patterns matching, laying hens, organic egg production systems, ozone

## Abstract

In Italy, organic egg production farms use free-range housing systems with a big outdoor area and a flock of no more than 500 hens. With additional devices and/or farming procedures, the whole flock could be forced to stay in the outdoor area for a limited time of the day. As a consequence, ozone treatments of housing areas could be performed in order to reduce the levels of atmospheric ammonia and bacterial load without risks, due by its toxicity, both for hens and workers. However, an automatic monitoring system, and a sensor able to detect the presence of animals, would be necessary. For this purpose, a first sensor was developed but some limits, related to the time necessary to detect a hen, were observed. In this study, significant improvements, for this sensor, are proposed. They were reached by an image pattern recognition technique that was applied to thermografic images acquired from the housing system. An experimental group of seven laying hens was selected for the tests, carried out for three weeks. The first week was used to set-up the sensor. Different templates, to use for the pattern recognition, were studied and different floor temperature shifts were investigated. At the end of these evaluations, a template of elliptical shape, and sizes of 135 × 63 pixels, was chosen. Furthermore, a temperature shift of one degree was selected to calculate, for each image, a color background threshold to apply in the following field tests. Obtained results showed an improvement of the sensor detection accuracy that reached values of sensitivity and specificity of 95.1% and 98.7%. In addition, the range of time necessary to detect a hen, or classify a case, was reduced at two seconds. This result could allow the sensor to control a bigger area of the housing system. Thus, the resulting monitoring system could allow to perform the sanitary treatments without risks both for animals and humans.

## 1. Introduction

### 1.1. Housing System Management in Eggs Production Farms

Housing system management is a key factor for the welfare of hens and the productivity of egg production farms. Different indexes have to be monitored and maintained within specific ranges. For example, the internal temperature of housing systems should not exceed the 16–24 °C range [1]; air humidity should be between 50% and 70% [1]; the concentration of atmospheric ammonia, caused by litter microbiological populations [2], should be limited to 20–25 ppm and not exceed this threshold for an interval of time of more than 8 h [3]; the concentration of carbon dioxide, caused by hens and heating systems [4], should be limited to 3000 ppm and this threshold should only be surpassed for intervals of time lower than 8 h [5]; the concentration of dust, that in different types of housing system can reach values of 6.5 mg/m^3^ and 80 mg/m^3^ (for respirable and inhalable dust, respectively) [6] should be limited to the lowest value that is achievable.

Through a correct ventilation, in terms of cycles and intensities, the indexes above reported can be monitored and controlled. In fact, the temperature can be maintained within a specific range of values, the air humidity can be reduced and the quality of air can be improved [7]. At the same time, the concentrations of atmospheric ammonia and bacterial population can be limited [7]. However, a decreased air humidity level could also bring about some other problems: higher concentrations of dust could be observed and higher heating costs could be incurred during the winter periods [1]. Thus, other procedures to be adopted in addition with those already used for the housing system management of egg production farms, could be useful. Ozone treatments could be one of these additional procedures. They could allow improving the air quality, limit odors, reduce levels of atmospheric ammonia and bacterial load [8]. The application of these treatments has been already investigated both for egg industry [9,10,11] and for egg production farms [12]. It has been proven that a concentration of ozone of at least 1 ppm, is necessary to perform an effective bactericidal procedure but, in the same time, its toxicity has been demonstrated both for animals and humans [13,14]. Therefore, to evaluate and use these treatments in a real farming system, it is mandatory to avoid that hens and workers be exposed to this chemical element [14].

### 1.2. Housing System Designs

Nowadays, in eggs production farms, hens can be housed in enriched cages or in free-range systems without cages. In this latter case, animals can use all the space available [15] and their welfare may be improved. Free-range systems can also provide an outdoor area. With this additional space, a further improvement of the hens’ welfare may be achieved because a lower animal density can be observed in the housing system during the daytime; resources may be more accessible and more chances, to carry out spatial behaviors can be provided for hens [16].

Dedicated free-range systems are generally adopted in organic egg production farms. These systems have a particular design that is the result of the adoption of regulations that these farms have in common. In general, it is required to: (a) have a flock of no more than 500 hens; and (b) provide an outdoor area, that can be partially covered, where plants, grasses, and abundant space per single hen must be available. In these free-range systems it has been proven that the number of hens, that could be outside the housing system at any given time can be up to 30–40% of the whole flock [16]. Furthermore, by using some specific devices (such as, providing feeders in the outdoor area and pop-holes that allow hens to move from the housing system to the outdoor area in only one direction for a short time during the day) the number of hens in the outdoor area could increase and probably includes the whole flock.

In these kinds of housing systems, daily ozone treatments could be possible allowing one to achieve an improvement of the farm management. High concentrations of ozone (1 ppm, and above) could be used, when the hens are in the outdoor area, and a good efficiency could be reached [17]. At the same time, the housing system could be opened again, to hens, only when concentrations are low, and no longer dangerous, allowing one to ensure low risks for the animals. Of course, to reduce the workers’ risks, the execution of these procedures should to be controlled by an automatic monitoring system and a sensor, able to detect the presence of animals in the housing system before the start of ozone treatments, as necessary in order to not expose the hens to this toxic agent.

### 1.3. Systems for the Monitoring Laying Hens

Many systems have been developed for the monitoring of laying hens using different technologies. In studies focused on the behavior of hens, one of the most adopted is radio frequency identification (RFID) [15,18,19,20,21]. For this a transponder, is fastened on an animal’s leg or wing that is able to transmit a number when interrogated by a reader, connected to an antenna. The number transmitted is unique, therefore, each hen can be identified. More than one antenna can be used (and positioned in a different place of the housing system) and each transponder can be read, many times, by each antenna. The movements of each animal, inside the housing system, can be recorded linking the information provided by the transponder and the antenna that has made the reading. Of course, the accuracy of the resulting monitoring system depends on the number of antennas installed. These monitoring systems have been developed, basically, to perform research activities. In production applications, they would be inadequate because: (1) all the hens of the flock need a transponder to be identified; (2) a lot of antennas would be necessary to control the whole area of the housing system; and (3) some transponders could be lost by hens and they should be immediately substituted in order to maintain the monitoring system operative. Therefore costs, system complexity and required management procedures would result to be expensive, not easy and time consuming.

For the detection of laying hen behaviors another technology that has been evaluated requires the use of sensors that must be worn by animals [22,23,24]. These sensors create a wireless network with some nodes that are fixed. These nodes are radio beacons. Collecting data from these nodes, the position of a hen in the housing system can be calculated and recorded. Using this technology, the resulting monitoring system can overcome some of the limits reported above because less components could be necessary for the controlling of a big area. However, in production farms, also this technology could show some problems. In fact, the sensors worn by hens need a power supply that can limit the monitoring activity when carried out for a long time. Furthermore, each hen has to wear its sensor in order to be detected. As a consequence, for a large flock, operative costs could also result excessive in this case.

Video surveillance is another technology that has been considered, many times, for the development of monitoring systems for poultry [1,25,26,27,28,29,30,31]. For example, Aydin et al. [26] tested an automatic monitoring system in order to measure the activity levels of broiler chickens with different gait scores. Five chickens were placed in six cages on the basis of a gait score given by human experts. Images were collected by a camera, mounted perpendicular to the floor and above the center of the cages. Images were analyzed by a dedicated software application that allowed to calculate, for each cage and over the time of observation, an activity index considering the intensity pixel changes between the collected frames. Dawkins et al. [27] evaluated the behavior of commercial broilers in order to find a significant relationship between the general movements of the whole flock and the welfare of chickens, measured in terms of rate of mortality, skin damage (hock burns) and poor gaits in walking activity. For this purpose, web-cameras were installed in some chicken houses and analysis of the optical flows were performed. These analysis allowed detection of the changes of pixels’ brightness in different parts of each frame. As a consequence, flow velocities were calculated and information about the behavior of animals were recorded and analyzed. Kashiha et al. [28], in a study on welfare of broilers reared in a commercial housing system, discussed a method for measuring a distribution index using a real-time monitoring system. A set of cameras were installed to acquire images from the house interior and a dedicated software application was used to calculate the index during the time of observation. However, in all these studies, video surveillance was generally used to collect data about the behavior of a group of animals. Therefore, a monitoring system based on this kind of elaboration would not be able to reach a good accuracy in the detection of a single animal. On the contrary, using an image pattern recognition technique applied to video surveillance, better results could be reached in the monitoring of hens activities.

Image pattern recognition allows one to detect a model in a picture. It is generally adopted in industrial processes to perform quality control of production cycles even though many other examples can be found in a lot of applications of machine vision. To perform a pattern recognition, a template must be defined. It is a model of the physical object that has to be searched in an image. One or more objects can be detected in the same picture even though they are rotated, partially distorted and/or they have different dimensions. The most important factors that can affect the results of a pattern recognition are the template selection and the image pre-processing definition (that are always application-dependent).

In poultry research, image pattern recognition tools have been investigated by many authors, with positive results [29,30,31]. For example, Nakarmi et al. [29] developed a system based on a 3D vision camera and a grid made by twenty antennas installed in an experimental pen floor. The system was used to collect images and RFID data during the monitoring tests. In a following phase, off-line, image processing procedures were performed in order to identify and track animals. The system allowed the authors to investigate the behavior of hens and to analyze the effects due by different housing designs and management schemas. Wang et al. [30] evaluated different software algorithms to track a laying hen within a flock of hens. Tests were carried out in the laboratory and an initial step, necessary to identify a rectangle that included the target to track, was required. The results showed that an algorithm, based on the Hybrid Support Vector Machine model, could be a valid tool in order to study the behavior of hens without the need of additional sensors, such as RFID transponders. Kashiha et al. [31] investigated the performance of image analysis in the monitoring of laying hens. An experimental cage, made up by four interconnected compartments, was used for laboratory tests and controlled by four cameras (i.e., one for each compartment). Acquired images were elaborated, off-line, using a pattern recognition technique. An ellipse was used as template for the detection of a hen in a compartment of the cage. When compared with human observations, the image processing system showed good hen detection results and allowed to collect data about the hen’s navigation between the compartments of the experimental cage. However, all the studies above cited were mainly focused on assessing the behavior and/or the welfare of hens. They were carried out in a laboratory, using dedicated cages for the housing of hens and/or specific experimental conditions. Furthermore, the resulting monitoring systems often required significant processing, performed off-line after have acquired some video recordings. Therefore, evaluations of a monitoring system in farm conditions (i.e., without the use of dedicated devices and/or set-ups such as cages, lights, etc.), able to perform elaborations in real time (i.e., able to classify a case giving immediately a result) with a good accuracy also for few hens can be considered as still lacking.

A partial answer to this need was given by our research group when it tested a monitoring system based on the use of a dedicated sensor for hen detection [8]. The sensor used thermografic images acquired from the room interior of a housing system through a thermo-camera, mounted perpendicular to the floor. Each image was evaluated, in real-time, by a software algorithm that counted the number of pixels with a higher temperature than a defined threshold. If the resulting value was higher than a specific reference, a hen was considered detected. This reference was proportional to the mean thermografic imprint of a hen. However, to achieve a good accuracy in the detection task, the resulting monitoring system was set-up to evaluate ten images before classifying a case. As a consequence, the “monitoring window” (i.e., the range of time of the monitoring task) was divided into “monitoring slots” and each monitoring slot, as a result of the selected set-up, required twenty seconds to classify a case (since each thermografic image was acquired every two seconds). This result, itself, was not negative because a sensitivity of 97.9%, and a specificity of 94.9%, were reached in the hens’ detection. Nevertheless, it could be a limit for the system use considering the field of view (FOV) shown by the sensor. This value didn’t allow one to control all the housing system area. A possible solution this issue could be the movement of the sensor around one or two of its axes (i.e., through rotations or movements parallel to the floor). In this way, a different area of the housing system could be controlled in a monitoring slot but to complete a full scan of the housing system, in a short time and in a reliable way, a short monitoring slot would be necessary. To reach this goal, different (and more advanced) elaborations of acquired thermografic images would be necessary.

In this study we present a further improvement of the sensor for the monitoring of laying hens. To achieve better detection performances, advanced elaborations of acquired thermografic images were developed. They mainly consisted in the application of techniques of image pre-preprocessing, pattern recognition and post-processing of acquired data. The final target of the study was to reduce the time necessary to classify a case (i.e., the interval of time of a monitoring slot) in order to reach a better control of the housing system. This should allow, in a future step of our research, to study and perform (automatically and daily) ozone treatments in a safe mode and consequently to achieve a possible reduction of levels of atmospheric ammonia and bacterial load in egg production farms, with a particular focus on those that produce organic eggs.

## 2. Materials and Methods

### 2.1. The Experimental Case

**Figure 1:**
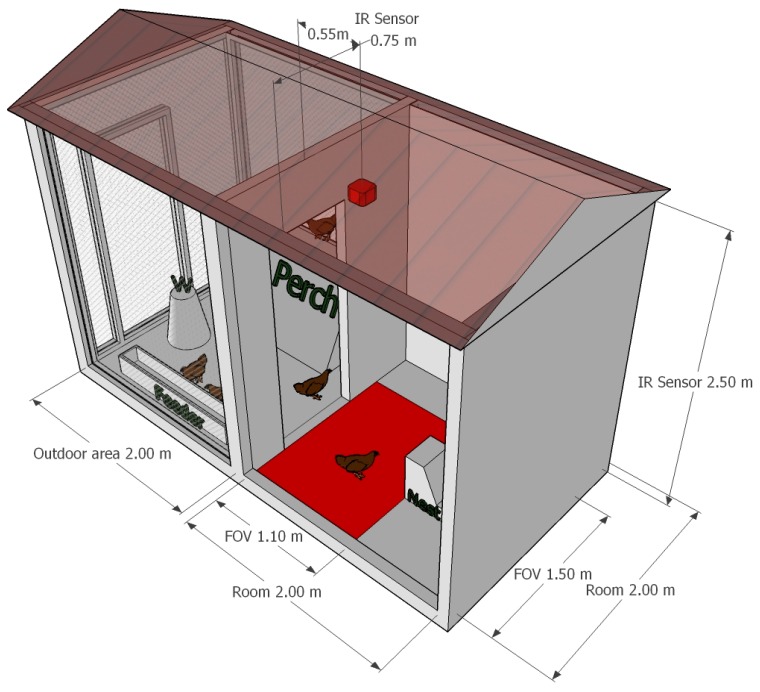
The picture shows the set-up of the experimental housing system and developed detection sensor. The water dispenser, that is represented with the symbol “W”, and the other main components of the housing system are reported. In the closed room, drawn in red, are shown: the detection sensor, mounted on the ceiling of the room, and its field of view (FOV) highlighted on the floor of the room. Finally, the dimensions of the housing system, of the sensor position and of its FOV, are also reported.

In order to set-up and to evaluate the sensor, for the monitoring of laying hens, an experimental housing system, without cages, was used. It included a covered outdoor area of 2.0 m × 2.0 m (H × W), a closed room, with equal sizes, one nest (of 30.0 cm × 45.0 cm × 45.0 cm (W × D × H)), a perch, a feeder, and a water dispenser (Figure 1). The experimental housing system provided a total amount of floor space equal to 8 m^2^ and the outdoor area provided a total floor space, for each hen, of 0.57 m^2^. This value was affected by the size of flock involved in the experiment (seven laying hens) that was decided according to the articles that defines the minimum standards for the protection of laying hens in the European Directive 1999-74-EC (4.1.c, 4.1.e and 4.4). The seven laying hens were randomly selected among animals that were reared (already in a group) on a commercial organic egg farm located in a region of Northern Italy-Lombardy). They were Institut de Sélection Animale (ISA) brown and they were housed, in the housing system used for the tests, a month before the start of the experiment approximately at the age of 18 weeks.

The sensor for the monitoring of laying hens was installed in the closed room of the housing system, 2.5 m above the floor. The sensor was positioned immediately after the room entrance and pointed directly downwards. The scope of the sensor was to detect the presence of hens, inside the closed room, during some specific intervals of time. Its field of view (FOV) was of circa 110.0 cm × 150.0 cm (H × W) and lower than the inner area of the closing room. As a consequence, a limitation of the hosing system floor space was necessary in order to allow, at the sensor, the monitoring of all the space available for hens.

### 2.2. The Layout of the Detection Sensor

A commercial thermografic camera (Thermo-GEAR-G120, AVIO, Nippon Avionics Co., Ltd., Tokyo, Japan) was the main component of the detection sensor under study. It was an uncooled (microbolometer) focal plane array detector that had a resolution in pixels of 320 × 240. It had an accuracy of ±2 °C; a sensitivity of 0.04 °C (at 30 °C) and sizes of 21.20 cm × 7.50 cm × 13.80 cm (H × W × D). As many other commercial thermo-cameras, it had an analog video output in order to be easily connected to an external monitor.

**Figure 2:**
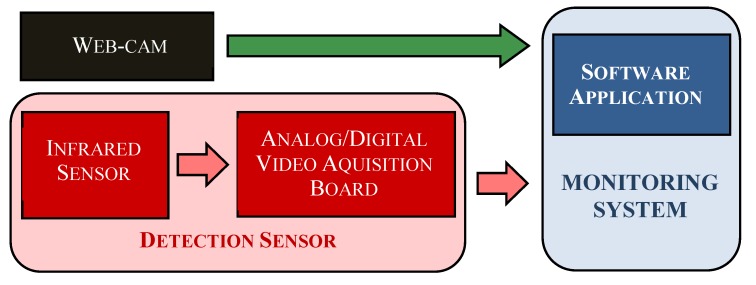
Components and connections of the experimental monitoring system. In the figure, it is also reported a commercial web-cam. This component was added to the monitoring system in order to collect, during the experiments, images of hens reared in the housing system. These images were used to evaluate the performance achieved by the detection sensor under study.

An acquisition board, for the analog/digital video conversion (ROXIO, Santa Clara, CA, USA), was also a part of the detection sensor. It received, through the analog video output of the thermo-camera, the grey-scale thermografic images acquired and it provided them, in a digital format, to a system for the monitoring of laying hens to which the sensor was linked (as it is shown in Figure 2). 

### 2.3. The Layout and the Tasks of the Experimental Monitoring System

**Figure 3:**
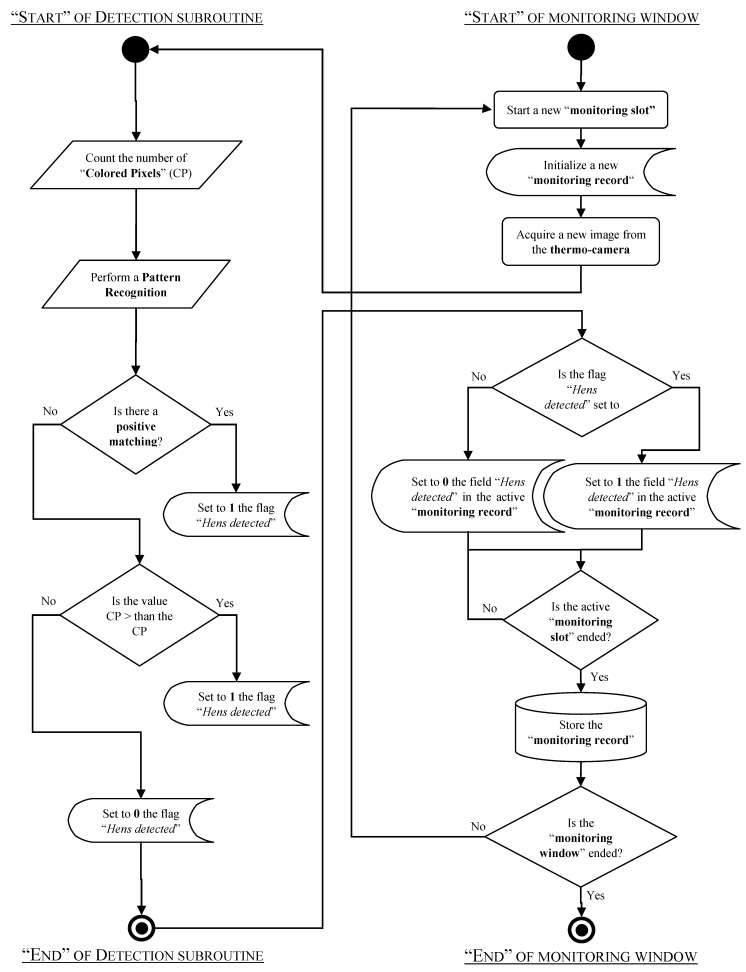
Flow diagram of the software application of the monitoring system.

For the set-up of the detection sensor and to quantify its accuracy, a monitoring system was developed. It was principally composed by a software application, developed using: NI LabVIEW^®^ 8.5 (National Instruments, Austin, TX, USA), NI Vision Acquisition Software^®^ 2009, and NI Vision Development Module^®^ 2009. Figure 3 shows a flow diagram that describes the tasks performed by the software application.

As reported in the flow diagram of Figure 3, when the “monitoring window” is active [32], a new “monitoring slot” starts and a new “monitoring record” is created [33]. In the steps that follow, the algorithm controls if at least one hen is detected. This control is done within a monitoring slot. When the monitoring slot is ended (i.e., after two seconds) the active monitoring record is closed and it is stored using an external memory. This storing is done by a text log-file that also includes: the time stamp of the slot start; the time stamp of the slot end; and some other data about the set-up of the detection sensor. When this step is concluded, a new “monitoring slot” is started and a new “monitoring record” is created. This tasks continue, with the same sequence, until the “monitoring window” is finished (i.e., when the monitoring system is switched-off).

**Figure 4:**
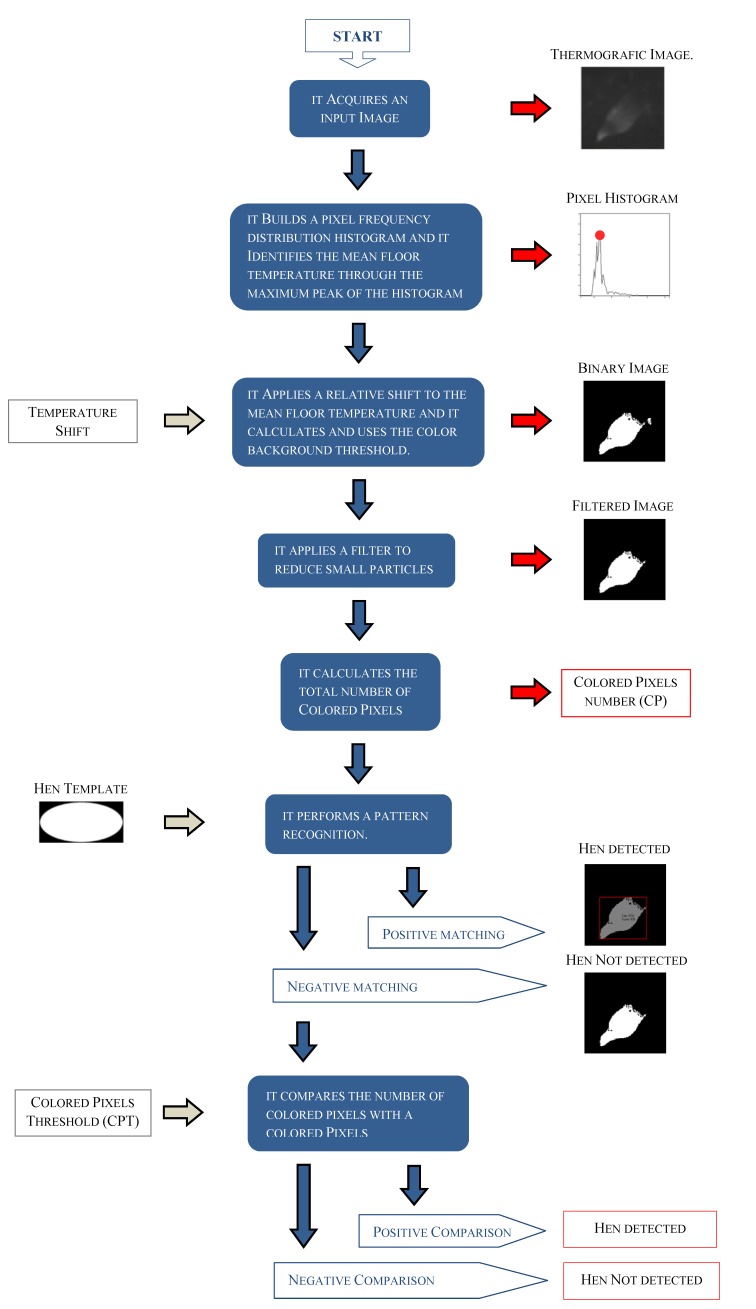
Flow diagram of the elaborations performed by the detection subroutine. The procedures of image pre-processing, pattern recognition and post-processing of acquired data are explained in detail. Each blue rectangle describes an elaboration performed by the subroutine which is linked to the following step of elaboration through a blue arrow. For some specific elaborations, with a grey arrow is highlighted an input while with a red arrow is shown an output.

In order to detect a hen, the software application used a specific subroutine. This subroutine performed some elaborations on each grey-scale image acquired by the thermo-camera (Figure 4). In details:

- it built an histogram with the frequency distribution of pixel intensities;
- since the histogram generally has a normal distribution, where the mean value corresponds to the background intensity, and considering that in a thermografic image to each intensity corresponds a specific measured temperature, it calculated the mean floor temperature of the closed room;
- it applied a defined shift to the mean floor temperature calculated and it converted the resulting value in a grey-scale color value (i.e., the “Background Color Threshold” or BCT);
- it applied the BCT to each grey-scale colored pixel in order to build a binary image (where a color is given to all pixels that overcame the threshold);
- it applied an image filter to reduce the number of small “particles”;
- on the resulting image, it calculated the total amount of “Colored Pixels” (CP);
- on the resulting image, it performed a pattern recognition using a defined “hen template”;
- in case of positive result, it set-up to 1 the flag “Hens Detected” (HD);
- in case of negative result, it compared the CP value with a “Colored Pixels Threshold” (CPT), that is proportional at the “visible” area of one hen (i.e., the case most restrictive);
- when the value CP overcame the value CPT, it set-up to 1 the flag HD;
- in the remaining cases, it set-up the flag HD to 0.

For the detection of a hen in a thermografic image, a pattern recognition procedure was performed by the software application subroutine. This procedure evaluated the Normalized Cross-correlation between the image acquired and the “hen template” previously set-up [34,35,36]. The Normalized Cross-correlation is a specific case of correlation that can be defined according to the following Equation (1):

(1) $$C\left(i,j\right)=\overset{V-1}{\underset{x=0}{\sum }}\overset{U-1}{\underset{y=0}{\sum }}t\left(x,y\right)p\left(x+i,y+j\right)$$

where *p*(*x*, *y*) is the image evaluated of sizes W × Z; *t*(*x*, *y*) is a template of sizes *U* × *V* with *U* ≤ W and *V* ≤ Z; *i* = 0, 1… W − 1; *j* = 0, 1,… Z − 1; and the summation is calculated considering the overlapping between the image *p* and the template *t*. In other words, the correlation procedure requires to move the template *t* within the image *p* and it calculates the value *C*(*i*, *j*) considering the region where the template *t* overlaps the image *p*. As a consequence, each pixel of the template *t* is multiplied for the corresponding pixel of the picture *p*, that it overlaps. Each result is summed over the all pixels of template *t*. The point where the function *C*(*i*, *j*) is maximum, is the place of the image evaluated where the template *t* is found.

However, the correlation procedure can be sensitive to pixel intensity changes of the images evaluated. To overcome this sensitivity, a normalized cross-correlation can be used, as it was done in this study, according to the following Equation (2):

(2) $$R\left(i,j\right)=\frac{\sum_{x=0}^{V-1}\sum_{y=0}^{U-1}\left[t\left(x,y\right)-\bar{t}\right]\left[p\left(x+i,y+j\right)-\bar{p}\left(i,j\right)\right]}{{\left\{\sum_{x=0}^{V-1}\sum_{y=0}^{U-1}{\left[t\left(x,y\right)-\bar{t}\right]}^{2}\right\}}^{\frac{1}{2}}{\left\{\sum_{x=0}^{V-1}\sum_{y=0}^{U-1}{\left[p\left(x+i,y+j\right)-\bar{p}\left(i,j\right)\right]}^{2}\right\}}^{\frac{1}{2}}}$$

where: $\bar{t}$ (that is calculated only one time) is the mean value of the pixel intensities of the template *t*; and $\bar{p}$ is the mean value of the pixel intensities of a region determined by the position of the template *t*. The resulting value (*R*) is always in a range from −1 to 1 and is not affected by any changes of the pixel intensities of both images (*p* and *t*). In addition, in case the object to detect has a different orientation respect the template *t*, the image template can be rotated and a new procedure can be carried out.

The monitoring system developed also collected images, in the visible spectrum, from the room interior. These images were used to check the detection results of the sensor under study and to evaluate its accuracy. Even though the thermo-camera adopted in the tests allowed to collect conventional pictures, a commercial web-cam was adopted to acquire these images (HAMA AC 150, Hama Gmbh & Co., Monheim, Germany). This technical solution was chosen in order to allow, in future experiments, the use of commercial thermo-cameras specifically developed for industrial applications that generally don’t have this feature. They are ideal for applications of monitoring because they are robust, cheaper and allow the image transferring on standard networks and interfaces (such as GigE Vision) by low-cost standard cables.

The web cam was installed close to the thermo-camera in order to have a similar FOV of the detection sensor. It worked with a sampling interval of one image for each cycle of the monitoring system (i.e., every two seconds). All collected images were saved as single snapshots using the “.bmp” extension and a pixel resolution of 320 × 240. The same images were also used to build video recordings of the housing system. These video recordings had: one frame per second as a picture sampling rate; an “.avi” extension; and were highly compressed using the filter “Microsoft Video 1”. Snapshots and video recordings were collected every day (from the start to the end of the “monitoring window”). To each image and video recording, dates and time stamps were added and used to name each file [37] that was stored in an external memory.

### 2.4. Procedures for Data Collection and Processing

Experimental tests were carried out for three weeks (from 7 November to 25 November). In the first five days the sensor was set-up while in the other days, its accuracy was measured.

When the sensor was set-up, a researcher performed a specific procedure that was repeated every day (i.e., five times in total). The procedure required to: (a) force to get out possible hens that were in the room; (b) randomly select one hen from the flock and move it inside the room; (c) close the room and (d) start the monitoring window (at 11.00 a.m.) in order to acquire experimental data for 15 min. After this interval of time, the hen in the room was forced to go out and for another 15 min a new monitoring window was started. After this further interval of time, the monitoring system was stopped and each limitation inside the room was removed. This procedure was adopted in order to have, in a well-controlled scenario (such as one hen or no hens in the closed room) enough data for the set-up of the detection sensor and it was repeated, for some days, in order to acquire enough data from different animals. In future experiments (or field applications), carried out with the same hardware components and the same experimental set-up, this procedure could be not performed as it was done in the other days of this experiment when a researcher only switched-on the monitoring system (between 11.00 a.m. and 11.30 a.m.) without perform any action on hens that were in the closed room.

Using a version of the software application able to work off-line, on “.bmp” files, the data stored in the first days of the experiment were analyzed. A sub-set of thermografic images (1472), within those stored (4416), were evaluated in two phases. In the first one, three different shifts, of the mean floor temperature, were investigated (i.e., the BCT that corresponded to a relative increment of 1, 2 and 3 °C) and the geometric features, of the templates to use in the following pattern recognitions, were identified. These temperature shifts were chosen considering our previous experiences [8] and what scientific papers suggest [38,39,40,41] as body temperature ranges that should result from a thermografic image of a hen. Considering each temperature shift, all “particles” that were in an image were identified and measured. For each particle, an equivalent ellipse, and rectangle, were identified and their minor and major axes, and long and short side, were calculated. However, in each shift of the mean floor temperature, not all data were considered. Since with a shift of 1 °C almost the whole hen can be “seen” in a thermografic image, while with the other shifts (2 or 3 °C) only the head of a hen can be well identified, in the case of a shift of one degree, only the data about equivalent ellipses were studied while in the other cases, only the data about equivalent rectangles were evaluated. As a consequence, a template of elliptical shape, and sizes of the resulting mean values, was identified for the temperature shift of 1 °C while for the other shifts, two different templates were selected. They had a triangular shape and sizes, in terms of bases and heights, equal to the mean values of the long and short sides of the equivalent rectangles calculated. In a following phase of the analysis, the same sub-set, of thermografic images, was elaborated. For each shift of the mean floor temperature investigated, a specific template was used for the pattern recognitions. The results obtained were analyzed considering the experimental set-up and video recordings acquired from the room interior. From these analysis, the results were classified as: true positive (TP), when one hen that was in the room was correctly detected; false positive (FP), when one hen was detected but no hens were in the room; true negative (TN), when it was correctly detected that no hens were in the room; and false negative (FN), when the sensor did not detected a hen that was in the room. When all these classifications were concluded, all the resulting data were organized in terms of: sensitivity, specificity, error rate, and accuracy according with the following Equations (3)–(6):

(3) $$\mathrm{Sensitivity}\left[\%\right]=\frac{\mathrm{TP}}{\mathrm{FN}+\mathrm{TP}}$$

(4) $$\mathrm{Specificity}\left[\%\right]=\frac{\mathrm{TN}}{\mathrm{FP}+\mathrm{TN}}$$

(5) $$\mathrm{Error}\;\mathrm{Rate}\left[\%\right]=\frac{\mathrm{FP}}{\mathrm{FP}+\mathrm{TP}}$$

(6) $$\mathrm{Accuracy}\left[\%\right]=\frac{\mathrm{TP}+\mathrm{TN}}{\mathrm{TP}+\mathrm{TN}+\mathrm{FP}+\mathrm{FN}}$$

The resulting values, that were obtained, were considered as the detection performance achieved by the sensor and the set-up, that allowed to reach the best performance, was used in the following tests.

During field tests, no specific procedures were performed by a researcher on the experimental housing system and/or on the hens of the experimental group. Every day, the monitoring system was switched-on at 11.00 a.m. At the same hour, a “monitoring window” started and collected for 30 min the experimental data. When the “monitoring window” was ended, a researcher switched-off the monitoring system and started to evaluate data acquired. He/she compared the log-file stored by the system and the video recording acquired from the closed room. On the basis of these comparisons, obtained results were classified in terms of: TP, FP, TN and FN; and subsequently in terms of sensitivity and specificity. In this way, the performance achieved by the detection sensor, under real working conditions, was calculated. During all the testing phases, a researcher checked daily the lenses of the thermo-camera and web-cam. When necessary, they were cleaned in order to ensure that high quality images were acquired.

## 3. Results

During the experiments, room temperature ranged between 5 to 13 °C showing an average value of 8 °C. In a first phase of elaborations, carried out on thermografic images acquired during the sensor set-up, three different shifts of the mean floor temperature (i.e., three BCT) were investigated and the geometric features of the templates, to be used in the following pattern recognitions, were identified. For each temperature shift, a different template shape was studied. In details, for the shift of one degree, an ellipse was selected while for the shifts of two or three degrees, a triangle was considered. The results that were obtained are reported in Table 1, in addition to their confidence intervals and significance of the means. These last values were calculated performing a *t*-Test of Student on collected data (procedure *t*-test, package *stats*, tool for statistical analysis: R—ver. 3.3.0 [42]).

In a following phase of elaborations, always performed on data acquired during the sensor set-up, sensitivity, specificity, error rate and accuracy, of the detection sensor, were calculated. For each floor temperature shift, a specific template was used in accordance with the results reported in Table 1. Obtained results of this phase of elaborations are shown in Table 2.

As reported in the table, the floor temperature shift that allowed the best performance, in terms of error rate and accuracy, was a shift of one degree. Therefore, in the experimental days that followed, the parameters that were used for the set-up of the sensor were: (a) a CBT that corresponded to a floor temperature shift of 1 °C; (b) a template that had an elliptical shape and dimensions of 135 × 63 pixels to perform the pattern recognitions; and (c) a CPT equal to 1736 pixels as required by the algorithm of the software application. This last value was defined considering the results of our previous experiments [8].

**Figure 5:**
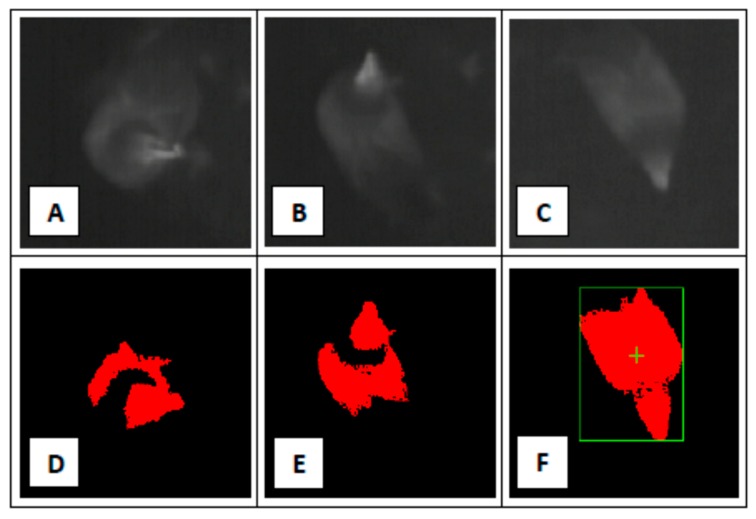
Pictures (**A**–**C**) are thermografic images of a hen acquired by the detection sensor during field tests. Pictures (**D**–**F**) are the results of the elaborations performed by the sensor considering as input the pictures (**A**–**C**). Picture (**F**) is an example of positive pattern recognition while pictures (**D**,**E**) are examples of negative pattern recognitions. Nevertheless, in picture (**E**), the software application counted 2089 colored pixels while in picture (**D**), the total amount of colored pixels was 1525. Therefore, at the end of the elaborations performed by the software application, the case of picture (**E**) was classified as positive while the case of picture (**D**) was confirmed as negative. Thus, in picture (**D**) is shown a false negative case while in pictures (**E**,**F**) are reported two examples of cases correctly classified as positive by the detection sensor developed.

In the final phase of the experiment, the accuracy of the detection sensor was evaluated through the sensitivity and specificity reached by the monitoring system under test. The log-files provided by the system, and the video recordings collected during the experiments from the room interior, were compared. Furthermore, a dedicated CPT was used to further investigate the cases that resulted negative in the pattern recognitions (Figure 5). The results that were obtained in these last evaluations are shown in the Table 3.

As reported in the table only in 118 cases (i.e., the 0.86% of the total number of cases evaluated) a hen that was in the room was not detected by the system during an active “monitoring slot” and the final accuracy, reached in a real working condition, was a sensitivity of 98.7% and a specificity of 95.1%. Examples of elaborations and classifications performed by the sensor are provided in Figure 5.

## 4. Discussion

The present study had, as its aim, the improvement of a sensor for the detection of a hen in a closed room of a housing system of an organic egg production farm. Furthermore, a reduction of the time necessary to perform the detection task, using infrared technology and an advanced processing of acquired data, was its specific aim. The performance recorded in the present field tests showed an improvement when compared with the values of sensitivity and specificity achieved in a first iteration of the sensor (98.7% and 95.1% vs. 97.9% and 94.9%-[8]). Furthermore, the monitoring slot was reduced from 20 s to only 2 s. This value could allow one to scan the whole housing system area, through rotations or movements of the sensor parallel to the floor, in a short time. In this way, possible limits of the sensor’ FOV should be overcome. However, this interval of time could be further reduced. In fact, the main result obtained in this study was to perform a detection task using only one thermografic image. This means that the working rate of the sensor could be increased up to the computational power of the hardware used to build the monitoring system. As a consequence, the monitoring slot could be further reduced to a value less than 2 s, allowing also a better managements of difficult cases due to a rapid movement of a hen inside the room of the housing system. Thus, on the basis of these results, the specific aim of the study should be considered as reached considering the use of a pattern recognition technique as the main part of the elaborations performed on thermografic frames collected.

Nevertheless, some false negative cases were found during this experiment. These cases were related to some unfavorable events that can happen during a monitoring slot, that were: (a) a hen was in a part of the room not well controlled by the sensor, such as the nest; and (b) the image processing performed was not corrected for the hen detection. For the first type of events, some possible improvements could be identified. The sensor’s FOV was principally affected by the dimensions of the room used for the experiments. In a bigger room, this value could be increased. The sensor could be mounted at a greater height and a thermo-camera, with a higher resolution and lens with a lower optical length, could be adopted. This set-up should increase the FOV of the sensor without losing the positive results achieved in these tests. In addition, other simple sensors could be coupled with the detection sensor in order to improve the monitoring system used in these tests. For example, a couple of photocells, mounted in the nests, could be adopted to detect a hen that is laying an egg. With these additional sensors, the monitoring system would be able to control all the areas generally included in a housing system of a commercial organic egg production farm. As a consequence, all the space available for animals could be better controlled.

The second type of events, that affected the accuracy of the detection sensor, were related to the transformations performed on thermografic images. These elaborations were mainly: a procedure of image pre-processing, a pattern recognition, and a post-processing of acquired data. For the pattern recognition, the selection of a template, in term of shape and sizes, is a key factor to reach good results. In this study, different shapes and sizes were studied considering the geometric features of the parts, of a hen, that were “visible” in the thermografic images evaluated. Obtained results showed as the best template to use, an ellipse with sizes of 135 × 63 pixels. However, some false negative cases were found. They were mainly due by the performing of the pattern recognition on animals, freely to move, and not on solid objects which shape is always well defined. A possible way to overcome this issue could be to allow a “scale” compensation. With this set-up, a greater number of particles could be identified by the template matching. Nevertheless, in the same time, an increased number of false positive cases could be found because not all particles, of a thermografic image, could be related to a hen or a part of its body (as required when a template that does not include the whole hen, it is used). Therefore, for this kind of applications, a different strategy has to be found and the merging of different types of elaborations might be a way to reach a better detection accuracy without increasing too much the execution time required. For this reason, in this study, image pre-processing and a post-processing of acquired data procedures were performed. The image pre-processing procedure, carried out on acquired grey-scale images, included: (a) calculation of a histogram with the frequency distribution of pixel intensities; (b) the use of a relative shift of the mean floor temperature; and (c) the adoption of an image filter. All these steps improved the effectiveness of pattern recognitions and the accuracy of the detection sensor. In fact, the histogram permitted us to compensate the automatic calibrations performed by the thermo-camera and the intrinsic error of the thermografic sensor in the measurement of an absolute temperature (i.e., ±2 °C or 2% of the measured value). The relative shift, of the mean floor temperature, allowed us to build binary images using a specific CBT for each thermografic image. The image filter allowed us to reduce the number of small particles that were not related to a hen or a part of its body. These possible visual artifacts can happen for different reasons. For example: (a) if a hen remains in the same place for few minutes, its feet can leave a thermal imprint on the floor that can become visible in the following images; (b) if an egg is laid outside the nest, having the same body temperature of the hen, it can be visible in many subsequent images (on the basis of the atmospheric temperature and of the relative shift that has been used); and (c) a specific point of the floor can has an higher temperature than the mean floor value and thus it may result in the image being evaluated. The results of the performed procedures were to obtain a more accurate set of binary images and less variability in the number of colored pixels. As a consequence, better shapes were available for the pattern recognitions and more accurate calculations, of the colored pixels of each image, were possible. This last result improved the performance of the post-processing phase that was carried out in order to reduce the number of false negative case and, therefore, improve the accuracy of the detection sensor developed.

More in general, the obtained results showed that image analysis and pattern recognition was a useful technical solution that allowed us to detect a hen, in a small monitoring slot, with performances in line with the results obtained in other studies. For example, Kashiha et al. [43] evaluated a method based on the use of pattern recognition to automatically identify marked pigs, in an experimental pen, in order to perform behavioral researches. For each of the four pens, where a group of ten pigs were reared, one camera was mounted. Video recordings were acquired during the tests and pattern recognition techniques were used to localize the pigs. Also individual identification of pigs was performed through a set of basic patterns that were painted on their backs. The tests showed that in 88.7% of the cases, a pig was automatically identified while in only 1% of the cases, a misidentification occurred (i.e., it was a false negative case). Poursaberi et al. [44] studied an algorithm to score different behaviors of turkeys confined in some cages. The aim of the research was to automatically collect data about the welfare of animals, especially during their transportation. One camera was mounted on each cage. Acquired images were evaluated by an image pre-processing procedure and a pattern recognition method. Having as reference the manual ability to classify the behavior performed the results of the study showed that the software application was able to correctly classify: 71% of standing, 83% of lying, 94% of wing flapping, and 100% of turning. Kashiha et al. [31] developed a monitoring system to detect hens’ movements within four compartments of an experimental cage. One camera was mounted on each compartment and acquired images were considered by a pattern recognition technique. Obtained results showed that compartment occupancies were correctly detected in 95.9% ± 2.6% of the cases and in only 4.2% ± 3.0% of the cases, a false occupation was recorded. More in general, the system proved that image analysis could be an alternative to manual video analysis when it is compared with the human ability to classify a case in the performing of monitoring activities.

Our results also confirmed that infrared technology is a valid way for the detection of hens inside a closed room. The good quality of acquired images was always guaranteed, even though no specific controls were applied to the light conditions inside the room. This feature pose, the developed sensor and the resulting monitoring system, as a device easily useable in a real commercial farm where an infrared sensor could bring, also, some other benefits. In fact, it has been proven by some authors that thermografic analysis could improve hens’ welfare [19,20,22]. Yahav et al. [38], for example, showed that systems for the climate control, performances of flocks, and welfare of animals, could be improved evaluating the physiological status of chickens through a thermografic camera that estimates the heat lost through radiation and convection. Baracho et al. [39], in a study aimed to identifying possible thermal distress of broilers reared in a commercial farm, used infrared technology in order to measure the broiler surface temperature as a result of the week of rearing and sector where the animals were housed. Ferreiera et al. [40], investigating broilers’ feeding, proved that a thermo-camera could be used to measure the variations of the surface temperature of broilers and to improve the productivity of the farm by the correct selection of feed energy levels. However, despite these positive results, the use of thermo-cameras in commercial farms remains scarce presumably for the high costs of this device [1]. Thus, a monitoring system based on infrared sensors could be an opportunity to bring in commercial farms the results already reached in the research that applied this technology. Monitoring systems able to analyze, and improve, the hens’ welfare could be developed. As a consequence, positive, and practical, improvements could be reached because the farm management, and the eggs production, should result to be increased. 

In this study, different floor temperature shifts were investigated during the set-up of the detection sensor. The shift of one degree showed to be the better set-up in order to achieve the best accuracy. This result was different from the result that we found in another study carried out during the summer time (i.e., a temperature shift of 3 °C [8]). Although the current elaborations, performed on thermografic images, were different from those curried out in the other study, we think that the present result confirms that the mean atmospheric temperature can affect the selection of the floor temperature shift to be applied. Thus, different temperature shifts should be identified, at least one for each season of the year, in order to achieve the best monitoring accuracy. Further experiments will be useful to validate this hypothesis and to build a formula in order to calculate, for each atmospheric temperature and/or season of the year, the right temperature shift to use.

## 5. Conclusions

The obtained results proved that infrared technology, and an advanced elaboration of acquired images, could allow to detect a hen in a closed room of an housing system of a commercial farm that produces organic eggs. Through techniques of image pre-preprocessing, pattern recognition and post-processing of acquired data, the monitoring slot, necessary to perform the detection task, could be reduced to 2 s. This should allow one to build a monitoring system able to control a bigger area of the closed room of the housing system. As a consequence, ozone treatments, that could reduce bacterial load and atmospheric ammonia levels, could be performed without risk for the reared hens or for workers.

## Figures and Tables

**Table 1:** Templates shapes and geometric features for each floor temperature shift investigated (i.e., 1, 2 and 3 °C). Values of the means, of the standard error (S.E.), of the confidence intervals and of significance of the means, obtained performing a Student’s *t*-Test procedure, are reported.

Floor Temperature Shift (∆*t*-°C)	Shape of the Template	Geometric Future	Mean and S.E. Values (Pixels)	Confidence Intervals at 95% (Pixels)	Significance
1	ellipse	major axis	134.8 ± 1.3	132.2–137.4	*p* < 0.01
1	ellipse	minor axis	62.6 ± 0.6	61.8–63.8	*p* < 0.01
2	triangle	base	34.9 ± 1.5	31.8–38.0	*p* < 0.01
2	triangle	height	16.4 ± 0.6	15.3–17.6	*p* < 0.01
3	triangle	base	17.7 ± 0.3	17.1–18.2	*p* < 0.01
3	triangle	height	11.2 ± 0.2	10.9–11.5	*p* < 0.01

**Table 2:** Values of sensitivity, specificity, error rate and accuracy that the developed detection sensor showed, during the set-up procedure, for each temperature shift investigated (i.e., 1, 2 and 3 °C). Furthermore, the geometric features of the templates used for the pattern recognitions are also reported.

Floor Temperature Shift (∆*t*-°C)	Sensitivity (%)	Specificity (%)	Error Rate (%)	Accuracy (%)	Shape and Geometric Features of the Template (Pixels)
1	80.4	92.5	8.5	86.4	ellipse (135 × 63)
2	43.3	89.0	20.1	66.0	triangle (35 × 16)
3	53.9	95.0	8.5	74.4	triangle (18 × 12)

**Table 3:** Accuracy showed by the detection sensor under test in field experimental conditions.

	Positive	Negative	Total
**True**	9108	4283	13391
**False**	222	118	340
**Total**	9330	4401	13731
